# Context-specific gene regulatory networks subdivide intrinsic subtypes of breast cancer

**DOI:** 10.1186/1471-2105-12-S2-S3

**Published:** 2011-03-29

**Authors:** Sara Nasser, Heather E Cunliffe, Michael A Black, Seungchan Kim

**Affiliations:** 1 Computational Biology Division, Translational Genomics Research Institute, 445 N. Fifth Street, Phoenix, AZ, USA; 2 Breast and Ovarian Cancer Unit, Computational Biology Division, Translational Genomics Research Institute, 445 N. Fifth Street, Phoenix, AZ, USA; 3 Department of Biochemistry, University of Otago, New Zealand; 4 School of Computing Informatics and Decision Systems Engineering, Arizona State University, Tempe, AZ, USA

## Abstract

**Background:**

Breast cancer is a highly heterogeneous disease with respect to molecular alterations and cellular composition making therapeutic and clinical outcome unpredictable. This diversity creates a significant challenge in developing tumor classifications that are clinically reliable with respect to prognosis prediction.

**Results:**

This paper describes an unsupervised context analysis to infer context-specific gene regulatory networks from 1,614 samples obtained from publicly available gene expression data, an extension of a previously published methodology. We use the context-specific gene regulatory networks to classify the tumors into clinically relevant subgroups, and provide candidates for a finer sub-grouping of the previously known intrinsic tumors with a focus on Basal-like tumors. Our analysis of pathway enrichment in the key contexts provides an insight into the biological mechanism underlying the identified subtypes of breast cancer.

**Conclusions:**

The use of context-specific gene regulatory networks to identify biological contexts from heterogenous breast cancer data set was able to identify genomic drivers for subgroups within the previously reported intrinsic subtypes. These subgroups (contexts) uphold the clinical relevant features for the intrinsic subtypes and were associated with increased survival differences compared to the intrinsic subtypes. We believe our computational approach led to the generation of novel rationalized hypotheses to explain mechanisms of disease progression within sub-contexts of breast cancer that could be therapeutically exploited once validated.

## Background

Complex diseases such as breast tumors frequently have genomic mutations, translocations, and increased or decreased dosage of genes. The complex regulatory arrangements are further permuted, producing extreme heterogeneity in regulation and severe analytic complications. Such heterogeneity prevents existing methods, which often assume a certain level of homogeneity in samples, from learning underlying regulatory mechanisms from molecular measurements of tumor tissues. This inherent heterogeneity also generates a need for specialized therapeutic response, necessitating the development of models of breast cancer that can incorporate such heterogeneity.

Several landmark studies have shown that array-based expression profiling can provide insight into the complexity of breast tumors and can be used to 1) derive a molecular taxonomy for breast cancer, and 2) provide prognostic information better than standard assessment of clinical variables [1]. For example, genomic grade, or proliferation index is a strong predictor of outcome in estrogen receptor alpha (ER) positive disease. Another example is the 21-gene OncotypeDx assay (Genomic Health, Redwood City, CA) used to stratify ER positive patients into risk of recurrence groups following endocrine therapy. From seminal work published by Dr. Charles Perou [2] and others, classification methods have been, and continue to be, used to define “intrinsic” subtypes of breast cancer. These subtypes include Luminal A, Luminal B, Basal-like, HER2-enriched and normal breast-like, and are believed to represent distinct biological entities. Moreover, multiple studies have now confirmed that patient survival significantly differs with respect to intrinsic subtype.

A pathway-based classification of breast cancer shows that intrinsic gene expression signatures can be built using knowledge from pathway activity on previously known subtypes [3]. The aim of the study was to provide a functional interpretation of the gene expression data that can be linked to therapeutic options. The paper by Gatza et al. [3] indicates that the intrinsic subtypes can have further subgroups which may lead to much better understanding of each subtype. Recently, a subgroup of Basal-like tumors associated with poor prognosis has also been reported [4,5].

### Aim of this work

To improve the modeling and inference of regulatory mechanisms from such heterogeneous samples, a biologically based approach to sample and process stratification that models and learns context-specific regulations was proposed and developed [6,7]. The model hypothesizes that genomic (expression) regulation is comprised of two distinct types: *convergent regulation and divergent regulation*, the former representing a particular set of genes being modulated by different sets of regulators, and the latter indicating a given set of regulators modulating entirely different sets of genes in different cellular contexts. The model also assumes that when a cell maintains a specific cellular context, (i.e a phenotype) it tightly regulates a battery of genes. It is hypothesized that the set of genes under such tight regulation would show rather deterministic transcriptional activities. When the cell moves away from this cellular context or changes to a different cellular state, the behavior of the same set of genes will not appear as deterministic since their behavior is now under the control of various external agents. In this paper, we will illustrate, using the concepts of *conditioning* and *crosstalk*, that systematic inquiry of candidate genes can identify a set of cellular contexts where a set of genes is tightly regulated, and corresponding context-specific gene regulatory networks.

Genomic regulation of breast cancer subtypes may show several common traits, although they have several unique features that make them distinct. The contexts obtained from this approach can be further used to study the underlying biology of the individual subtypes, which can lead to a better understanding of the differences and similarities between the tumors.

In contrast to previous methods, we used an unsupervised method to identify biologically meaningful cellular contexts within breast cancer. Our motivation lies in modeling the heterogeneity of breast cancer with a context-specific approach.

## Results and discussion

The results section describes the data collection process, followed by the context analysis, phenotype and functional enrichment analysis and survival analysis.

### Breast cancer data collection and processing

Ten breast cancer Affymetrix HG-U133A microarray data sets were downloaded from the NCBI GEO data repository (http://www.ncbi.nlm.nih.gov/geo/). These cohorts contain distinct clinical and molecular features such as ER+/ ER-, PgR+/ PgR-, Grade and LN+ and LN- types. Table 1 lists the data sets along with the number of samples within each cohort. The data from all cohorts were combined and normalized together by RMA normalization. A 2-fold change was used to categorize genes as under-expressed, no change or over-expressed; thus generating a data with ternary values {-1, 0, 1}. The cohorts contain a total of 1,887 samples with some samples repeated in more than one cohort. After removing the duplicates, a total of 1,636 samples were obtained. Additionally, GSE 2603 contains some cell line data that was removed reducing the number of samples to 1,614.

**Table 1:** Breast cancer cohorts

GEO Accession No.	Sample Size
GSE3494 [21]	251†
GSE4922 [1]	289†
GSE2990 [22]	189
GSE1456 [23]	159
GSE7390 [24]	198
GSE11121 [25]	200
GSE12093 [26]	136
GSE2603 [27]	121‡
GSE5327 [28]	58
GSE2034 [29]	286

GEO Breast cancer cohorts containing 1887 samples were reduced to generate the 1614 sample dataset. † 248 overlapping between these cohorts were removed to retain unique samples. ‡ 22 cell lines were removed from this cohort keeping patient samples only

Many variables in the data sets have low variance and may not contribute to network learning. These variables with low variance across all samples were removed from the data sets. This also reduced the dimensionality of the data and made the network learning process computationally more tractable. Affymetrix probe sets were matched to *HUGO* gene symbols, probes matching to the same genes were combined by taking the median of the probes with Spearman’s correlation of 0.8. Probe sets with lower correlation values were discarded. After filtering at a variance of 0.14 and combining probes, we reduced the variable size to 5,023 highly variant genes.

### Context analysis

**Figure 1:**
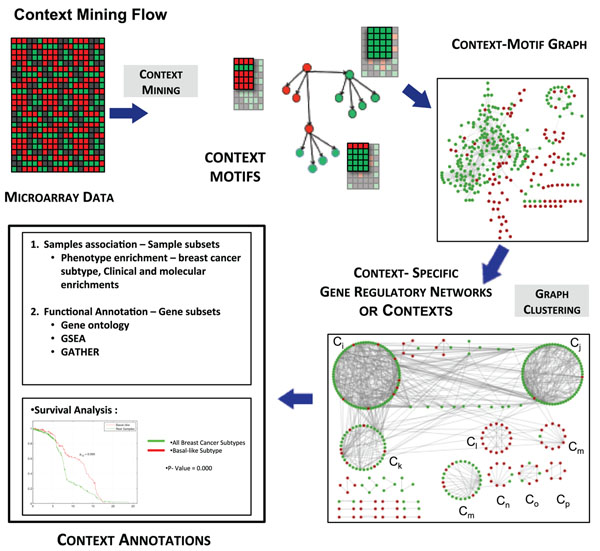
**Context-Mining process flow**. The process to analyze heterogeneous biological data to learn context-specific gene regulations is illustrated in this figure. We first identify context-motifs using crosstalk, conditioning and statistical p-value computations. Since some genes can be a driver in a context motif, but a passenger in other context motifs, these context motifs can be chained together to build a interaction graph. In this graph, each edge represents an interaction specific to certain subset of samples (context motif). We now use this property along with graph clustering to identify potential cellular contexts where we should see a set of interactions sharing significant numbers of samples in common. Once cellular contexts are identified, we annotate each context (which includes a subset of samples and a subset of genes) using gene enrichment, subtype enrichment, or survival analysis methods as described in the paper.

A context-specific gene regulatory network was generated for the data using a parallel implementation of the algorithm called ExPattern (available at http://sysbio.fulton.asu.edu/expattern). The steps involved in finding contexts from the breast cancer expression data is illustrated in Figure 1. A graph with context-motifs filtered at a statistical significance of < 0.05 after FDR correction was generated. A total of 1,466 context-motifs generated at this step were clustered using Markov clustering (MCL) [8] to obtain 189 clusters, which are referred to as ”contexts” henceforth in the paper. MCL was performed on the graph with an inflation of 3.0 to keep the granularity high, and connectivity was imposed within clusters, such that each context contained connected context-motifs only. Contexts with less than 80 samples (< 5% of total samples) may not convey meaningful results and thus were discarded, resulting in 41 contexts. Specificity of the contexts was measured by computing pairwise Jaccard distance between the contexts for both samples and genes [9]. The contexts had an average Jaccard distance of 0.96 for genes and 0.85 for samples, indicating that most of the contexts were well separated with little overlap. A summary of context analysis with respect to the number of associated samples and genes is given in Table 2.

**Table 2:** Contexts summary

Contexts	Samples	Genes	ER +/ ER-	PgR+/PgR-	LN+/LN-	Grade	Subtypes
C 89	1418	2	ER+	PgR+		Low	Normal, LumA
C 16	1330	16	ER+	PgR+		Low	Normal, Her2, LumB, LumA
C 75	1200	4					
C 34	1186	23			LN+		
C 68	1068	4					
C 40	1044	6			LN+	Low	LumA
C 57	824	7			LN+	High	Basal
C 73	805	3		PgR+	LN-	High	LumB
C 51	788	6	ER+	PgR+		Low	Normal, LumA
C 18	738	10	ER-	PgR-	LN+	High	Normal, Basal
C 67	731	4					
C 79	658	2	ER+	PgR+		Low	Normal, LumB, LumA
C 55	551	6			LN-	High	
C 49	549	6			LN-		
C 150	395	5	ER-	PgR-	LN+	High	Basal, Her2
C 126	336	2			LN-	Low	LumA
C 162	248	2	ER-	PgR-		High	Basal, Her2
C 134	234	4	ER-	PgR-		High	Basal, Her2
C 160	202	2	ER-	PgR-		High	Basal
C 48	188	27	ER-	PgR-		High	Basal
C 121	186	2					Normal
C 143	185	4	ER+		LN-	High	
C 147	175	3			LN-	High	LumB
C 168	154	5	ER-	PgR-	LN-	High	Basal
C 146	153	2			LN-	High	
C 110	152	5	ER-	PgR-		High	Basal
C 145	150	9	ER-		LN-	High	Basal
C 159	150	3	ER-				Basal
C 130	129	2	ER-	PgR-		High	Basal
C 124	128	2			LN-	High	LumB
C 131	126	2	ER+	PgR+		Low	Normal, LumA
C 28	121	10			LN+	Low	Normal
C 155	119	3	ER-			High	Basal
C 50	118	42	ER+				LumA
C 153	115	5					Normal
C 139	111	2					
C 104	95	5					
C 144	90	7			LN-	High	
C 22	86	31	ER-	PgR-	LN+	High	Basal, Her2
C 115	86	2	ER+	PgR+	LN+	Low	Normal, LumA
C 111	84	4	ER-	PgR-		High	Basal,

Results show contexts (ordered by number of samples) obtained after context-motif mining and MCL in column 1; contexts samples associated with a threshold 0.7 and specificity of 2 in column 2 and context genes in column 3. Context enrichments with clinical and molecular features (ER Status, PgR Status, Grade and LN Status) are shown in columns 4-7, selected after a statistical significance of < 0.05 (Low Grade =Grades 1 and 2; High Grade = Grade 3). Last column shows intrinsic subtype enrichments with LumA=Luminal A, LumB= Luminal B, Basal = Basal-like , Her2 and normal Tumors.

### Clinical characterization and subtype enrichment

**Figure 2:**
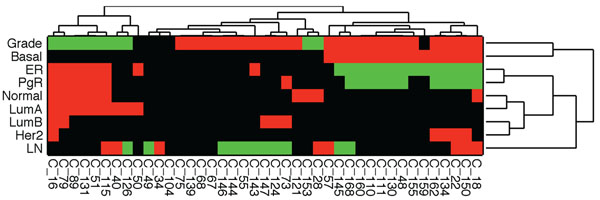
**Context clusters.** Hierarchical clustering of contexts with clinical and subtype enrichments

Following clustering, the contexts were analyzed for clinical and molecular marker enrichments. Additionally, intrinsic subtypes were also associated with contexts with statistically significant enriched subtypes. Clinical and molecular markers and intrinsic subtypes associated with each context are listed in Table 2. A reasonably large number of contexts showed enrichment for at least one subtype. The grouping of ER+ intrinsic subtypes (LumA, LumB and Normal) and ER- tumors (Her2 and Basal-like) was clearly evident with the context enrichment. Basal-like tumors associated with low survival, showed high grade consistent with previous studies of Basal-like breast cancer. Additionally, LumA and LumB types were enriched with more than one context and Basal-like tumors were enriched in several contexts. Average Jaccard distance of samples for LumA contexts is 0.75 and LumB context is 0.85. There were no overlapping genes between the LumA and LumB contexts. The average Jaccard distance of samples for Basal-enriched contexts was 0.84, indicating that these groups are highly distinct and may indicate subgroups of Basal-like tumors. Table 2 shows some contexts enriched with multiple intrinsic subtypes, and we studied this further by grouping contexts and intrinsic subtypes based on their co-enrichments, via hierarchical clustering. Enrichments were annotated with ternary values 1, 0, -1, indicating presence, absence and, in the case of some clinical features, presence of negative types. Clinical enrichments ER, PgR, LN status and Grade were encoded as “-1” for ER-, PgR-, LN- and Low grade tumors, respectively, and positive “1” for ER+, PgR+, LN+ and high grade tumors, respectively. Hierarchical clustering was performed using Hamming distance and clusters were chained with complete linkage. The result is shown in Figure 2, which indicates biologically relevant groups for subtypes and clinical features. For example, Basal-like tumors known to be associated with high grade are clustered with grade. Luminal A tumors group with Normal-like tumors and Luminal B group with Her2-like tumors. Additionally, correspondence between ER and PgR states is also observed in the clustering result.

### Functional annotation

Functional annotation on the contexts with gene sets from MSigDB revealed interesting results. The results validate the enrichment of the contexts with ER+ and ER- tumors, and gene sets pertaining to these characteristics were found. Context 16 an ER+ and Luminal-like enriched context showed significant enrichment with Luminal-like breast cancer gene sets (p-values: 6.00*E* – 12, 1.38*E* – 10, 1.07*E* – 08). Context 48, ER-, high grade, Basal-like context was enriched with ER- gene sets and with invasive breast cancer gene sets (p-values: 0.00*E* + 00). Context 168 (ER-, Basal-like context) showed enrichment with ER- breast cancer gene sets and with Basal-like breast cancer gene sets (p-values: 1.55*E* – 04, 3.32*E* – 06). Additional pathways for some selected contexts are included in the Supplement tables 1 - 7 (see Additional file 1 Supplement tables 1-7).

### Survival analysis

**Figure 3:**
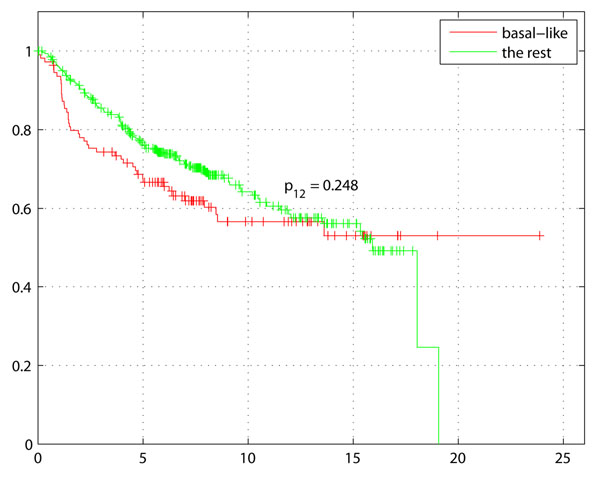
**Survival for all Basal-like samples.** Survival plot (in years) for all Basal-like tumors compared to rest of the tumors (all non Basal-like).

**Figure 4:**
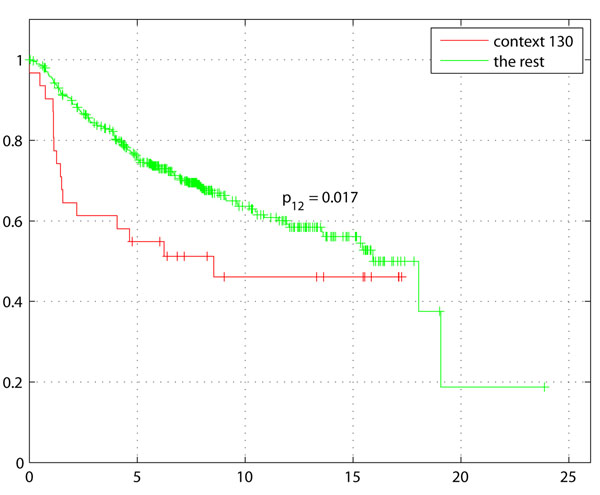
**Survival for Basal-like Context 130**. Survival plot (in years) for Context 130 enriched within a subgroup of Basal-like tumors shows poor survival compared to all Basal-like tumors. GATA3 which was under-expressed in this context was correlated with increased tumor size and estrogen and progesterone receptor negativity [20], confirming the poor survival indicated in this context.

**Figure 5:**
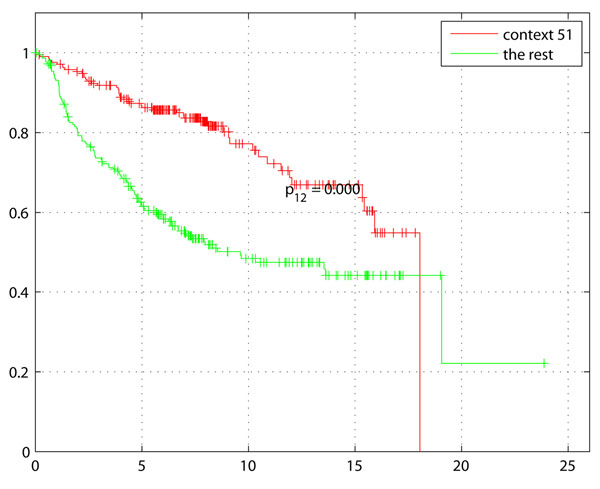
**Survival for ER+ context samples**. Context 51 enriched with low grade, ER+ tumors indicates expected increase in survival compared to rest of the tumors.

Survival analysis was performed on the 436 samples out of 1,614 with survival data (see Table 3)The Kaplan-Meier plot in Figure 3 with survival of Basal-like tumors, demonstrates the difference with rest of the tumors (non-Basal) with disease free survival (DFS) as the endpoint. The Kaplan-Meier plot of Basal-like enriched context 130 (genes: GATA3, INPP4B) in Figure 4 not only indicates shorter survival as expected for higher grade, ER- tumors but also a larger separation from the rest of the samples including other Basal-like tumors. Comparison of Figures 3 and 4 clearly indicates a potential sub-grouping within Basal-like tumors. Kaplan-Meier plot of Context 51 (genes: BUB1, DLG7, CENPA, MAD2L1, TTK, MCM10) ER+ tumors also indicates a better survival of ER+ tumors compared to rest of the samples (Figure 5).

**Table 3:** Context sample survival

Contexts	Samples with Survival Data	Median Survival	Rest Survival	p-value
C 89	364	7.00	5.60	0.3723
C 16	359	6.70	6.40	0.1284
C 75	366	7.00	5.90	0.9436
C 34	192	6.00	7.80	0.2202
C 68	289	6.70	6.60	0.7809
C 40	388	7.00	6.20	0.7053
C 57	115	5.90	7.30	0.4309
C 73	190	7.00	6.40	0.2099
**C 51**	214	7.60	5.70	**0.0000**
C 18	254	7.30	6.00	0.2651
C 67	236	7.30	6.30	0.6522
C 79	207	7.10	6.40	0.3360
C 55	183	7.30	6.40	0.2083
C 49	221	7.30	6.30	0.3516
C 150	137	7.10	6.50	0.7527
C 126	121	6.70	6.60	0.1025
C 162	71	7.50	6.40	0.2031
C 134	64	7.30	6.50	0.7523
C 160	75	7.70	6.40	0.7879
C 48	43	6.90	6.60	0.8853
C 121	12	5.90	6.90	0.3452
C 143	15	6.50	6.70	0.1870
C 147	8	7.10	6.60	0.5274
C 168	40	7.70	6.50	0.1569
C 146	6	6.70	6.70	0.1116
C 110	46	7.50	6.50	0.7452
C 145	1	5.8	6.7	1.0000
C 159	72	7.60	6.30	0.9455
**C 130**	31	6.00	6.70	**0.0166**
**C 124**	9	2.60	6.90	**0.0000**
C 131	11	7.40	6.60	0.4671
C 28	22	4.40	7.00	0.2096
C 155	57	7.50	6.40	0.9311
C 50	82	6.40	6.70	0.1115
C 153	29	7.10	6.60	0.9383
C 139	6	7.50	6.60	0.6071
C 104	6	6.40	6.70	0.7090
**C 144**	4	6.50	6.70	**0.0000**
C 22	32	7.40	6.50	0.9088
**C 115**	20	7.30	6.60	**0.0370**
C 111	27	7.90	6.50	0.3558

Survival analysis results: Contexts 51, 130, 124, 144 and 115 indicate statistically significant survival difference with the rest of samples.

### Discussion

Several contexts of biologic interest and potential translational potential were highlighted by this analysis that appear both expected, and novel. Context 51, indicative of ER-positive and PgR-positive, low grade, Luminal A and normal-like tumors, was significantly enriched for genes associated with cell cycle checkpoint regulation, specifically, the M phase of mitotic cell cycle (BUB1 MAD2L1 TTK). As would be expected for ER+ low grade tumors, which tend to exhibit lower levels of proliferation, this context correlated with an increase in median survival (Figure 5* p* = 7.8997*e*10^–8^). Context 89 shared the same enriched subtypes as context 51, and contained just 2 genes from the same family, MAGEA3 and MAGEA6. The potential utility of MAGEA (Melanoma Antigen family A) proteins as a biomarker of the presence of micrometastases and circulating tumor cells has been previously reported [10]. We noted that in this instance, the MAGEA genes were associated with tumors that typically have better outcome. It is interesting to speculate whether analysis of MAGEA proteins in circulating breast tumor cells or micrometastases may enhance prognostication in stage III or IV breast cancer. This has not yet been studied. Contexts 57, 48 and 145 were three of several contexts associated with the Basal-like intrinsic subtype and high grade tumors, each with strikingly different apparent molecular underpinnings. Context 57 contained genes (e.g., TEK) suggestive of highly angiogenic Basal-like breast tumors [11]. This tumor context includes positive lymph node status and a decrease in median survival (5.9 vs 7.3 months). In contrast to context 57, context 48 which contained 27 genes, was significantly associated with cell cycle, with no significant difference in prognosis, perhaps due to low numbers of tumors with survival data within this context. Context 130, a Basal-like context has under-expression of GATA3 which is in concordance with previous studies of Basal-like subgroup, ’claudin-low’ with poor prognosis and more refractory to chemotherapy [5] . Lastly, context 145, again a Basal-like context of high tumor grade and ER negative status contained genes associated with deregulated secretory pathways and mechanisms of docking and fusion of vesicles to target membranes. The gene PSENEN in this context codes for a gamma secretase and is known to play a role in intramembranous processing of proteins such as Notch, a key mediator of cell-fate, tissue patterning and morphogenesis. PSENEN protein is required for Notch pathway signaling [12] and Notch signaling is deregulated in breast cancer [13]. Interestingly, Prat et al have also identified a subtype of Basal-like breast cancer with Notch-associated signaling deregulation [4]. Additional genes in context 145 (such as, MAP3K2) point to deregulated MAPK, NFkB and PKC signaling, all of which are oncogenic in breast cancer and have been reported to be linked to Notch deregulation. As Notch signaling is emerging as an attractive therapeutic target in breast and other cancers [13], this context was of particular interest. There was only one sample with survival data in context 145 for prognostic evaluation, however the trend was an association with poor survival. Context 124 is consistent with the low survival of patients with LumB tumors (*p* < 1.1897*e*10^–7^). The above summarizes a sampling of contexts which highlight important unanswered questions in translational breast cancer research. Validation of these hypotheses to explain mechanisms of disease progression within sub-contexts of breast cancer have a potential to be therapeutically exploited.

There are a number of well characterized commercially available breast cancer cell lines that mimic various stages of breast cancer progression and biologic characteristics (including luminal A, HER2 enriched, Basal-like, invasive, non-invasive, metastatically competent, etc). Genes of interest identified as part of a specific context can be experimentally manipulated *in vitro* using breast cancer cell lines that match the phenotypic and/or molecular context of interest. Techniques commonly used to manipulate an individual gene within a viable cell line include RNA interference technology, which specifically eliminates expression of any specified target gene, use of target-selective drugs, or use of exogenous DNA gene expression constructs, which are engineered to introduce and express a specific gene of interest in a cell. The biological and molecular consequences of manipulating expression of a specific gene can then be measured using cell-based and/or molecular techniques to validate a computationally predicted hypothesis. Once verified, this information can be leveraged to develop more accurate prognostic or predictive biomarkers for clinical application.

## Conclusions

This papers demonstrates the application of context-specific gene regulatory networks to identify biological contexts within heterogeneous breast cancer data over many samples. This large sample set identifies a finite number of contexts linked with intrinsic subtypes and clinical parameters. Diagnosis of intrinsic subtype is an important step that aids the prognostics for breast cancer. Our analysis of intrinsic subtype gene expression signatures is consistent with previous findings of individual cohort molecular profiling studies. Previously established intrinsic subtypes show different mechanisms indicating a possibility of further grouping of the intrinsic subtypes. Distinct contexts of Basal-like tumors confirm the existence of subgroups within Basal-like tumors as reported in previous studies. The contextual drivers identified for these subgroups can help explain the molecular aspects for the groups. Several new genes were found driving some contexts that have not been previously reported to be associated with known subgroups within these subtypes. Functional annotation of the genes associated with contexts also revealed different characteristics associated with each subgroup that can be biologically validated to define signatures for the groups.

## Future work

The results of the experiments in the paper provide a promising approach to finding gene and clinical signatures associated with intrinsic subtypes within breast cancer. Nevertheless, biological validation of the genes involved is necessary and can strengthen the signatures for each context. Future directions include testing the results on a independent data set to group subtypes.

## Methods

In this section, we first describe succinctly an approach to infer context-specific gene regulatory networks [7], [14], [15], a metric to associate samples with appropriate context, and then describe statistical tests to identify pathways and clinical phenotypes that are enriched in context.

### Inferring context-specific gene regulatory networks

Previously, we developed a method to infer context-specific gene regulatory network from gene expression data [7], [14], [15]. In this section, we describe the method that we have further refined since then, by introducing context-motif mining, followed by graph-clustering of context-motifs to infer contexts and corresponding context-specific gene regulatory networks.

#### Mining context-motifs

Given a gene *g_k_* as a driver gene and a condition defined by a subset of samples *M_j_*, the algorithm uses probabilistic measures to identify a set of genes, i.e. passenger genes, that show a coherent molecular pattern within the condition. We define this set of genes, one or more of which function as drivers and the others as passenger genes, *context-motif.* Formally, a context-motif is represented as *C_i_* = (*G_i_, Y_i_, S_i_, M_i_*) where *G_i_* represents a set of driver genes, *Y_i_* the possible states of the genes (an example would be -1, 0, +1 for a ternary quantized data set), *S_i_* a set of passenger genes, and *M_i_* the set of samples under which coherent expression is observed.

Coherence of expression pattern and its specificity are measured by two statistics, *conditioning* (*δ_k_*) and *crosstalk* (*η_k_*), as given in Eqs. 1 and 2, which determine if a gene *k* displays a cohesive expression pattern specific to a cellular context regulated by Y=1, where *X_k_* is state of driven genes.

*δ_k_***=** 1 – *P*(*X_k_***=** 1 | *Y* = 1),   (1)

*η_k_* = *P*(*X_k_***=** 1 | * Y* ≠ 1)     (2)

Conceptually, conditioning measures the lack of transcriptional coherence in the condition of interest and crosstalk measures the specificity of coherence. This is based on the property that, cell deviates from its regulatory behavior under environmental changes or, in this study, more specifically, the presence of tumor. A change in the cellular context can be used to condition a subset of samples.

Since both crosstalk and conditioning parameters are estimated from observations, the statistical significance (p-value) of these parameters is computed by hypergeometric probability, to determine whether the patterns found in this case are not by chance.

The algorithm to identify all potential context-motifs interrogates every gene in the data set as a potential driver gene (*G_i_*) by being in a specific state (*Y_i_*) across a subset of samples (*M_i_*) and to find all corresponding passenger genes (*S_i_*). As we test every gene in the data set, we also estimate the statistical significance (*p*-value) of identified context-motif *C_i_* via permutation test and multiple testing correction by Storey’s false discovery rate (FDR) [16].

Once the context-motifs are identified with statistical significance, each context is considered to manifest regulatory relationships between the driver genes and corresponding passenger genes, i.e *G_i_* → *g* ∊ *S_i_*, specific to *M_i_* with *G_i_*(drivers) conditioned on a specific state *Y_i_* = *y_i_*. A driver *g_j_* in context-motif *C_j_* could be a passenger in another context-motif *C_i_*, conditioned by *g_i_*. When such implicit driver-passenger relationships *g_i_* → *g_j_* are added together, a set of context-motifs identified from a given data set can be represented as a graph. The context-motif-specific gene-gene interactions represented in a graph can be further analyzed as described below to reveal context-specific gene regulatory network.

#### Contexts and context-specific gene regulatory networks

The graph described above consists of several hundreds (or thousands) of context-motifs and thousands of gene interactions, and each interaction is specific to certain subset of samples. Hence, this graph might be sub-divided into sub-networks based on its topological structure, and each sub-network might be associated with subset of samples. We utilize a clustering technique for graph, specifically, Markov clustering, as described in Ramesh et al. [8,15].

*Markov clustering* (MCL) is an unsupervised graph clustering algorithm that simulates the flow in a graph using the notion of random walks. If a random walk visits a node in a cluster, it would be likely to visit several other members of the cluster before leaving the cluster [8].

The algorithm consists of two alternating operations; *expansion* and *inflation* to simulate the flow. Graph expansion is identical to taking the power of a matrix using matrix multiplication, which homogenizes the flow across different regions of the graph. The second operation, inflation, is mathematically equivalent to taking the Hadamard power of a matrix followed by scaling. Simply, the graph is denoted by a matrix of transition probabilities and expansion computes random walks by assigning probabilities with all pairs of nodes, since there are more paths within a cluster than between clusters the probabilities will be higher within a cluster. To maintain the stochastic property of the matrix, inflation re-scales the columns. Thus, the inflation parameter controls the granularity of the clusters. We use an implementation of Markov clustering based on the algorithm proposed by van Dongen [17].

### Sample-Context association

Contexts obtained from clustering consist of quite a few context-motifs each of which is individually represented by a set of variables (genes) and conditions (samples). We developed a method to aggregate all the samples assigned to the context-motifs in a context and to determine if a sample can be specifically associated with the context with statistical significance.

Formally, let *N* be the number of samples and *k_i_* the number of samples in a context motif *C_i_*. Now let ***C*** be a context made of {*C*_1_, *C*_2_,…, *C_m_*}*.* In a simple approach, the samples for the context cluster can be assigned by combining all the samples in every context-motifs:(3)

However, some samples could be present in only one or two context-motifs and may not represent the overall context. Hence, we use a metric to evaluate samples that are consistently present across majority of the context-motifs to systematically associate samples to context. Let *C*^{^*^j^*^}^ ⊂ ***C*** denote the subset of *C* in which the sample *s_j_* is included. Then, we define a likelihood that sample *s_j_* belongs to ***C***, considering the fact that each context motif *C_i_* consists of different number of samples, as:(4)

where *s_j_* ↦ *C* indicates *s_j_* is assigned to ***C***, and(5)

to compensate the different sample size associated with each context motif. It’s easy to see 0 ≤ *L*(*s_j_* ↦ ***C***) ≤ 1, where *L*(*s_j_* ↦ ***C***) = 0 indicates no appearance of the sample in any context motif, while *L*(*s_j_* ↦ ***C***) = 1 indicates the presence of the sample in every context motif. *K* is used to control how favorably one wants to consider context-specificity of sample membership to a given context. The higher the *K*, the more context-specific the sample membership is.

### Enrichment analysis

#### Intrinsic subtypes of breast cancer

A method, Single Subtype Predictor (SSP), for individual class classification developed by Hu et al. [18] was used to classify tumors from the 1,614 samples into five *intrinsic* subtypes. The algorithm uses the expression of 306 “intrinsic genes” across 315 samples of known subtypes to define a “centroid” (expression profile) for each subtype (available at https://genome.unc.edu/pubsup/breastTumor/). New tumors are then classified based on the expression profile of these 306 genes, with tumors assigned to the closest subtype centroid using Spearman rank correlation as a measure of distance. Probe sets from the Affymetrix data sets used here were mapped to the 306 genes in the intrinsic gene set, with median log base 2 intensities used when multiple probe sets matched a gene in the “intrinsic” list. The log-transformed expression data for each gene was then mean-centered within each cohort, before comparing them to the subtype centroid for classification.

#### Phenotype enrichment

Subsequent to clustering of contexts and associating samples to contexts, we study the phenotypic characteristics of each context. We use the intrinsic subtypes, as described above, such as Estrogen receptor (ER) status, Progesterone receptor (PgR) status, lymph node (LN) status and grade of the tumor, as phenotypes. Each of the phenotype determines certain characteristics of the tumor and can reveal therapeutic treatment options. Tumors contexts enriched with these phenotypes can provide interesting biological insights. Enrichment of contexts with a certain phenotype can be performed using hyper-geometric probability with multiple testing correction [16].

#### Functional annotation: gene set enrichment analysis

In addition to the phenotypic enrichments of a contexts, we also investigate the enrichment of biological functions in each context, using gene set associated with each context. The Molecular Signatures Database (MSigDB) consists of collections of gene sets such as Gene Ontology (GO) gene sets, gene sets for Biological Processes, pathway gene sets, curated sets, and computationally predicted gene expression neighborhoods underlying certain biological characteristics [19]. Genes can be annotated using a method called gene set enrichment analysis, which computes the enrichment of database gene sets with the genes found in the contexts. This method also uses hypergeometric test to measure the significance of the enrichment. A gene annotation tool GATHER was also used in for annotation of contexts (http://gather.genome.duke.edu/). The overall process of mining context-motifs followed by chaining context-motifs to obtain contexts can be illustrated in Figure 1. The process flow diagram also illustrates functional annotation processes for genes within the contexts and phenotype enrichment for samples belonging to each context.

## Authors' contributions

SN and SK participated in the design of the study. SN performed the data preparation and analysis. MB did the classification of the tumors into intrinsic subtypes. HC did the biological evaluation and wrote discussion section. The draft was initially prepared by SN and SK and was reviewed by HC and MB. All authors reviewed the final manuscript.

## Competing interests

The authors declare that they have no competing interests.

## Supplementary Material

Additional file 1 **Functional annotation for contexts** Functional annotation for selected contexts is provided as Supplement tables 1-7. Each table lists pathways or gene sets found to be enriched with genes from a context, size of the pathway or gene set, its description, amount of overlap and statistical significance. Click here for file
