# Supplementary material for: Context-specific gene regulatory networks subdivide intrinsic subtypes of breast cancer
Source: BMC Bioinformatics. 2011 Mar 29;12(Suppl 2):S3. doi: 10.1186/1471-2105-12-S2-S3 (PMC3073183; doi:10.1186/1471-2105-12-S2-S3)
Supplement: Additional file 1 — Functional annotation for contexts Functional annotation for selected contexts is provided as Supplement tables 1-7. Each table lists pathways or gene sets found to be enriched with genes from a context, size of the pathway or gene set, its description, amount of overlap and statistical significance. [file 1471-2105-12-S2-S3-S1.pdf]

Supplement tables

Functional annotation for the genes within contexts with gene sets from MSigDB

(http://www.broadinstitute.org/gsea/msigdb/index.jsp).

Supplement table 1 - Functional annotations for Context 16

Gene set name	Genes in gene set (K)	Description	Overlap (k)	k/K	p-value
Lien Breast Carcinoma Metaplastic vs Ductal Dn	110	Genes down-regulated between two breast carcinoma subtypes: metaplastic (MCB) and ductal (DCB).	8	0.0727	0.000000
Charafe Breast Cancer Luminal vs Mesenchymal Up	456	Genes up-regulated in luminal-like breast cancer cell lines compared to the mesenchymal-like ones.	8	0.0175	0.000000
Charafe Breast Cancer Luminal vs Basal Up	383	Genes up-regulated in luminal-like breast cancer cell lines compared to the basal-like ones.	7	0.0183	0.000000
Vantveer Breast Cancer ESR1 Up	175	Genes up-regulated in the luminal B subtype of breast cancer.	5	0.0286	0.000000

Supplement table 2 - Functional annotations for Context 48

Gene set name	Genes in gene set (K)	Description	Overlap (k)	k/K	p-value
Sotiriou Breast Cancer Grade 1 vs 3 Up	153	Up-regulated genes whose expression correlated with histologic grade of invasive breast cancer tumors: comparison of grade 1 vs grade 3.	14	0.0915	0.000000
Farmer Breast Cancer Basal vs Luminal	335	Genes which best discriminated between two groups of breast cancer according to the status of ESR1 and AR [Gene ID=2099, 367]: basal (ESR1- AR-) and luminal (ESR1+ AR+).	11	0.0328	0.000000
Blum Response to Salirasib Dn	343	Selected genes down-regulated in response to the Ras inhibitor salirasib [PubChem=5469318] in a panel of cancer cell lines with constantly active HRAS [Gene ID=3265].	9	0.0262	0.000000
Rhodes Undifferentiated Cancer	60	Genes commonly up-regulated in undifferentiated cancer relative to well-differentiated cancer, based on the meta-analysis of the OncoMine gene expression database.	6	0.1	0.000000
Naderi Breast Cancer Prognosis Up	37	Up-regulated genes in the breast cancer prognostic signature of 70 genes that significantly correlated with survival.	5	0.1351	0.000000
Poola Invasive Breast Cancer Up	304	Genes up-regulated in atypical ductal hyperplastic tissues from patients with (ADHC) breast cancer vs those without the cancer (ADH).	8	0.0263	0.000000
Vantveer Breast Cancer Metastasis Dn	107	Genes whose expression is significantly and negatively correlated with poor breast cancer clinical outcome (defined as developing distant metastases in less than 5 years).	6	0.0561	0.000000

Supplement table 3 - Functional annotations for Context 50

Geneset name	Genes in gene set (K)	Description	Overlap (k)	k/K	p-value
Reactome Tie2 Signaling	18	Genes involved in Tie2 Signaling	2	0.1111	0.000179
Reactome Signaling in Immune System	366	Genes involved in Signaling in Immune system	4	0.0109	0.000684
Cell Cell Signaling	400	Genes annotated by the GO term GO:0007267. Any process that mediates the transfer of information from one cell to another.	4	0.01	0.000952
KEGG Rig I like Receptor Signaling Pathway	71	RIG-I-like receptor signaling pathway	2	0.0282	0.002800

Supplement table 4 - Functional annotations for Context 51

Gene set name	Genes in gene set (K)	Description	Overlap (k)	k/K	p-value
Sotiriou Breast Cancer Grade 1 vs 3 Up	153	Up-regulated genes whose expression correlated with histologic grade of invasive breast cancer tumors: comparison of grade 1 vs grade 3.	5	0.0327	0.000000
Mitotic Cell Cycle Checkpoint	21	Genes annotated by the GO term GO:0007093. A signal transduction-based surveillance mechanism that ensures accurate chromosome replication and segregation by preventing progression through a mitotic cell cycle until conditions are suitable for the cell to proceed to the next stage.	3	0.1429	0.000000
Cell Cycle Checkpoint GO 0000075	47	Genes annotated by the GO term GO:0000075. A point in the eukaryotic cell cycle where progress through the cycle can be halted until conditions are suitable for the cell to proceed to the next stage.	3	0.0638	0.000000

Supplement table 5 - Functional annotations for Context 130

Gene set name	Genes in gene set (K)	Description	Overlap (k)	k/K	p-value
Streicher LSM1 Targets Up	40	Genes up-regulated in MCF10A cells (breast cancer) by expression of LSM1 [Gene ID=27257] off a letiviral vector.	2	0.05	0.000002
Haddad T Lymphocyte and NK Progenitor Up	83	Genes up-regulated in hematopoietic progenitor cells (HPC) of T lymphocyte and NK (natural killer) lineage.	2	0.0241	0.000007
Doane Breast Cancer ESR1 Up	114	Genes changed in breast cancer samples according to the ESR1 [Gene ID=2099] status: ER positive vs ER negative tumors.	2	0.0175	0.000014

Supplement table 6 - Functional annotations for Context 145

Gene set name	Genes in gene set (K)	Description	Overlap (k)	k/K	p-value
Protein Metabolic Process	1199	Genes annotated by the GO term GO:0019538. The chemical reactions and pathways involving a specific protein, rather than of proteins in general. Includes protein modification.	4	0.0033	0.000141
Secretion by Cell	114	Genes annotated by the GO term GO:0032940. The regulated release of a substance by a cell.	2	0.0175	0.000374
Membrane Organization and Biogenesis	133	Genes annotated by the GO term GO:0016044. A process that is carried out at the cellular level which results in the formation, arrangement of constituent parts, or disassembly of membranes inside and surrounding the cell.	2	0.015	0.000509
Positive Regulation of Catalytic Activity	157	Genes annotated by the GO term GO:0043085. Any process that activates or increases the activity of an enzyme.	2	0.0127	0.000707
Cellular Protein Metabolic Process	1086	Genes annotated by the GO term GO:0044267. The chemical reactions and pathways involving a specific protein, rather than of proteins in general, occurring at the level of an individual cell. Includes protein modification.	3	0.0028	0.002140
Macromolecular Complex Assembly	276	Genes annotated by the GO term GO:0065003. The aggregation, arrangement and bonding together of a set of macromolecules to form a complex.	2	0.0072	0.002160
Cellular Macromolecule Metabolic Process	1100	Genes annotated by the GO term GO:0044260. The chemical reactions and pathways involving macromolecules, large molecules including proteins, nucleic acids and carbohydrates, as carried out by individual cells.	3	0.0027	0.002220
Cellular Component Assembly	294	Genes annotated by the GO term GO:0022607. A cellular process that results in the assembly of a part of the cell.	2	0.0068	0.002440

Supplement table 7 - Functional annotations for Context 168

Gene set name	Genes in gene set (K)	Description	Overlap (k)	k/K	p-value
Benporath ES Core Nine Correlated	100	Breast cancer compendium: 100 transcription regulators showing most correlated expression with the 9 'embryonic stem cell' transcription factors that are preferentially and coordinately overexpressed in the high-grade, ER-negative breast cancer tumors.	2	0.02	0.000155
Smid Breast Cancer Basal Up	676	Genes up-regulated in basal subtype of breast cancer samles.	4	0.0059	0.000003
